# Enterotoxigenic *Bacteroides fragilis*: A Possible Etiological Candidate for Bacterially-Induced Colorectal Precancerous and Cancerous Lesions

**DOI:** 10.3389/fcimb.2019.00449

**Published:** 2020-01-17

**Authors:** Samin Zamani, Reza Taslimi, Akram Sarabi, Seyedesomaye Jasemi, Leonardo A. Sechi, Mohammad Mehdi Feizabadi

**Affiliations:** ^1^Laboratory Sciences Research Center, Golestan University of Medical Sciences, Gorgan, Iran; ^2^Department of Microbiology, School of Medicine, Golestan University of Medical Sciences, Gorgan, Iran; ^3^Division of Gastroenterology and Hepatology, Imam Khomeini Hospital, Tehran University of Medical Sciences, Tehran, Iran; ^4^Department of Microbiology, School of Medicine, Tehran University of Medical Sciences, Tehran, Iran; ^5^Department of Biomedical Sciences, University of Sassari, Sassari, Italy

**Keywords:** enterotoxigenic *Bacteroides fragilis* (ETBF), colorectal cancer, *bft* gene, adenoma, precancerous lesions

## Abstract

Enterotoxigenic *Bacteroides fragilis* (ETBF) produces *Bacteroides fragilis* toxin (BFT), which is associated with acute diarrheal, inflammatory bowel disease, and colorectal cancer (CRC). In experimental models, ETBF has been shown to contribute to colon carcinogenesis. The present study was conducted to investigate mucosal colonization of ETBF in the colon to find a possible association between the presence of ETBF and precancerous and cancerous lesions. The mucosal biopsies of involved sites were obtained from 68 patients with precancerous and cancerous lesions and 52 healthy controls (HC). The samples were cultured on Bacteroides Bile Esculin agar. Then, specific primers were designed to detect *B. fragilis* and *bft* gene using quantitative real-time PCR, and the possible links of ETBF with clinicopathological characteristics was evaluated. Also real-time PCR was performed to detect the *bft* gene subtypes. *Bacteroides fragilis* was detected in 51% of the patients and 48% of HCs cultures. The 16SrRNA gene was found to be present in 63 and 81% of the patients and HCs' samples, respectively. Moreover, the *bft* gene was detected in 47 and 3.8% of the patients and HCs, respectively. Also, *B. fragilis* was significantly more abundant in the patients' samples compared to those of HCs. In the patient group, higher odds ratio (OR) of ETBF was significantly associated with serrated lesions and adenoma with low-grade dysplasia. The *bft1* gene was the most prevalent subtype of *bft gene*, followed by the *bft2* gene. This was the first study in Iran to demonstrate increased positivity of ETBF in patients with precancerous and cancerous lesions. In this study, the *bft* gene was found to be associated with CRC, especially in the patients with precancerous lesions and initial carcinogenic lesions. Moreover, the results suggest that mucosal BFT exposure is common and could be a risk factor and a screening marker for developing CRC.

## Introduction

*Bacteroides fragilis*, which is found in the gastrointestinal flora of the humans and livestock, is an anaerobe bacterium. It is one of the prominent human commensal and one of most common isolated Bacteroides from the clinical samples which causes diarrhea, peritonitis, intra-abdominal abscesses, sepsis and endogenous purulent infections (Sears et al., 1995, 2014).

It has been shown that *B. fragilis* prevents intestinal inflammatory diseases in animal with colitis due to production of immunomodulatory molecule polysaccharide A (PSA) that induces an anti-inflammatory immune response in intestinal tissue (Mazmanian et al., 2008; Lee et al., 2018). The pathogenicity of *B. fragilis* is due to several factors, including the capsule, outer-membrane proteins (OMPs), and special enzymes that comprise a 20 kDa metalloprotease termed *Bacteroides fragilis* toxin (BFT) (Sears et al., 1995, 2014).

BFT-producing *B. fragilis*, enterotoxigenic *B. fragilis* (ETBF), has been known as a cause of diarrheal disorders in humans and animals (Myers et al., 1987; Purcell et al., 2017). ETBF is known as a risk factor for inflammatory bowel disease (IBD) and it is present in the stool and biopsy specimens of the patients (Prindiville et al., 2000; Basset et al., 2004; Zamani et al., 2017). BFT expression is revealed to cleave extracellular domain of E-cadherin, which is a major structural constituent of the zonula adherens and is responsible for cell adhesion. Moreover, BFT can activate B-catenin signaling and induce IL8 secretion in colonic epithelial cells (Wu et al., 1998). A study indicated that following BFT treatment of HT29/C1 cells, loss of membrane associated E-cadherin initiated the nuclear localization of ß-catenin, which induced c-myc translation and led to persistent cellular proliferation (Wu et al., 2003). The potency of BFT and its influence on gastrointestinal epithelial structure and physiology suggest that the presence of ETBF may contribute to chronic colonic diseases, including oncogenic transformation, intestinal inflammation, chronic colonic dysfunctions, and colorectal precancerous and cancerous lesions (Wu et al., 1998, 2003; Sears et al., 2014).

Colorectal cancer (CRC) is one of the most prevalent cancers worldwide and comprise 9% of all cancers and it is the fourth cause of cancer-related deaths worldwide (Sears et al., 1995; Ferlay et al., 2015). However, there is a significant difference between countries in age standardized incidence of this cancer; the incidence rate in the US and European countries is more than 25 times of that of African and Asian countries. The morbidity and mortality rate of CRC has been reducing in the last years because of enhanced screening tests that can detect colorectal modifications at earlier stages and improvements in medication and procedures (Boyle and Langman, 2000; Rafiemanesh et al., 2016).

CRC is one of the most common cancers in Iran and is the third most common cancer among Iranian men (8.1–8.3 per 100 000 populations) and the fourth most prevalent cancer among Iranian women (6.5–7.5 per 100,000 populations) (Moghimi-Dehkordi et al., 2008; Kolahdoozan et al., 2010). The risk factors of CRC are obesity, sedentary lifestyle, high fat diet, low vegetables and fruit diet, smoking, alcohol abuse, and non-steroidal anti-inflammatory drugs (NSAIDs). Chronic inflammation, IBD, polyps, adenoma, and dysplasia cause changes to colon cells and make them cancer prone (Boyle and Langman, 2000; Johnson et al., 2013).

The incidence and mortality of CRC is decreasing in developed Western nations, while its incidence is increasing among both sexes during the last decades in Iran due to lifestyle and dietary changes. Another reason for this decrease may be the increase in the number of facilities and improvement in equipment and technology, as more people refer to health care facilities for screening, while in the past, a person might have had a cancer and even have died, but the cancer went undiagnosed due to lack of equipment and facilities (Malekzadeh et al., 2009; Siegel et al., 2014).

In this study, the frequency and abundance of ETBF in biopsy samples of the patients with CRC and precancerous conditions were compared to those of the individuals with no personal or familial history of colorectal disease to investigate the association between the presence of BFT and tumor development.

## Materials and Methods

### Patients and Specimens

In this case control study, 120 mucosal biopsies were collected from Iranian patients with precancerous and CRC condition (*n* = 68) and control group (*n* = 52) using colonoscopy. Patients with precancerous (**Serrated lesions**, **Adenoma** include Low-grade Dysplasia: **LGD** and high-grade dysplasia: **HGD**) and cancerous conditions (Colorectal Cancer: CRC) who referred to Imam Khomeini hospital in Tehran between March 2015 and Jan 2017 were selected to participate in this study. The Ethics Committee of Tehran University of Medical Sciences approved the study protocol. Also, informed consent was obtained from all participants. All patients were diagnosed based on clinical symptoms as well as histologic and radiographic standards, which showed typical features with special distribution (Swiderska et al., 2014). All data on age, gender, and type of lesions were retrieved from patients' records. All of the patients that enter in the study diagnosed at the time of colonoscopy and chemotherapy didn't start for treating them.

During the same period, 52 healthy controls, with no personal or familial history of diagnostic colorectal disease, whose age and gender matched with those of the patients were included in the study as controls. A recent history of diarrhea and IBD was an exclusion criterion for controls. None of the individuals who took part in this study used any antibiotics or probiotics in the last 3 months. All the specimens were maintained in the sterile container comprising thioglycollate medium (Merck, Germany) and transported to the laboratory in an anaerobic condition for immediate handling. Also, 2 mucosal biopsies were collected from each patient for culture and DNA extraction.

### Bacterial Culture

Two glass homogenizers were used for mechanical disruption and homogenization for all the biopsies; then, they were cultured on Bacteroides Bile Esculin agar (BBE) (Himedia Laboratories Pvt. Ltd, India) medium. In this study, Anoxomat system (MART Microbiology Drachten, Netherlands) was used to provide gaseous atmospheric conditions for anaerobes; then, the plates were incubated in an anaerobic chamber at 37°C for 72 h. Moreover, *B. fragilis* was confirmed using real-time PCR method.

### DNA Extraction

DNA was extracted directly from the biopsy tissue using RTP® Mycobacteria Kit (Invitek, Berlin, Germany). The optical density (OD) of the extracted DNA was determined at 260 nanometres. Then, DNA was preserved at −20°C for subsequent analysis and real-time PCR.

### Real-Time PCR

The sequences of the *bft* and 16S rRNA gene were regained from the Gene bank. The primers and probes were designed using primer 3 plus (http://www.bioinformatics.nl/cgi-bin/primer3plus/primer3plus.cgi). All the primers and probes used for the detection of all *bft* gene types had been designed in our previous study (Zamani et al., 2017). In order to detect the *bft* gene subtypes, the real-time PCR was performed as previously described (Merino et al., 2011).

The genomic DNA of ETBF strain D-134 and RIGLD clone 1 were used as positive controls (Rashidan et al., 2018). The negative control was PCR TaqMan master mix with distilled water instead of DNA.

Standard DNA was prepared for amplification and the number of molecules of the template per gram was calculated by the defined formula (Zamani et al., 2017). The standard curve for *bft* and 16S rRNA gene was assessed using each primer and probe with a 10-fold serial dilution of *B. fragilis* DNA samples, corresponding to 10^1^-10^6^ mean value per gram of biopsies.

According to the standard curve and *y*-intercept, samples that did not show the fluorescent signal earlier than the Ct of 38 were determined as negative. Also, samples that produced fluorescence of a Ct value ≤ 10 were diluted. The efficacy of the real-time PCR was determined as E = 10(−1/slop) – 1 (Zamani et al., 2017).

After optimization of standard curves, the dilution series was put in each amplification run. Real-time PCR tests were evaluated using LinGene K Real Time PCR tool (Bioer, Hangzhou, PR, China). All the tests were done in a volume of 25 μL (Zamani et al., 2017). To ensure quality, all experiments were repeated for a second time independently and the means were reported.

To check the specificity of the PCR and the expected size of the product, the primers were applied in a conventional PCR and the amplicons were run on the agarose gel. Moreover, specificity of the positive amplified fragments was proved by sequencing. The PCR product was sent to the South Korea's Macrogen Corporation for sequencing. Sequencing results were analyzed with Chromas 2.6 software. Then, all the sequences were blasted in NCBI database (https://blast.ncbi.nlm.nih.gov/Blast.cgi). In all of the sequenced isolates the percentage of similarity to the related genes (16s rRNA gene and *bft* gene) present in the NCBI database were more than 95%.

### Statistical Analysis

The results were compared using Fisher's exact, Chi square, and Mann–Whitney tests. *P* < 0.05 was considered as statistically significant. Also, mean values ± Std. error of mean (SEM) were calculated for *B. fragilis* and ETBF. Data were analyzed using SPSS 13.0 statistical software.

In this study, conditional logistic regression was used to assess odds ratios (OR) and 95% confidence intervals. In this design, the odds ratio is a consistent estimator of the rate ratio of CRC when exposed with ETBF vs. unexposed subjects.

## Results

**Figure 1:**
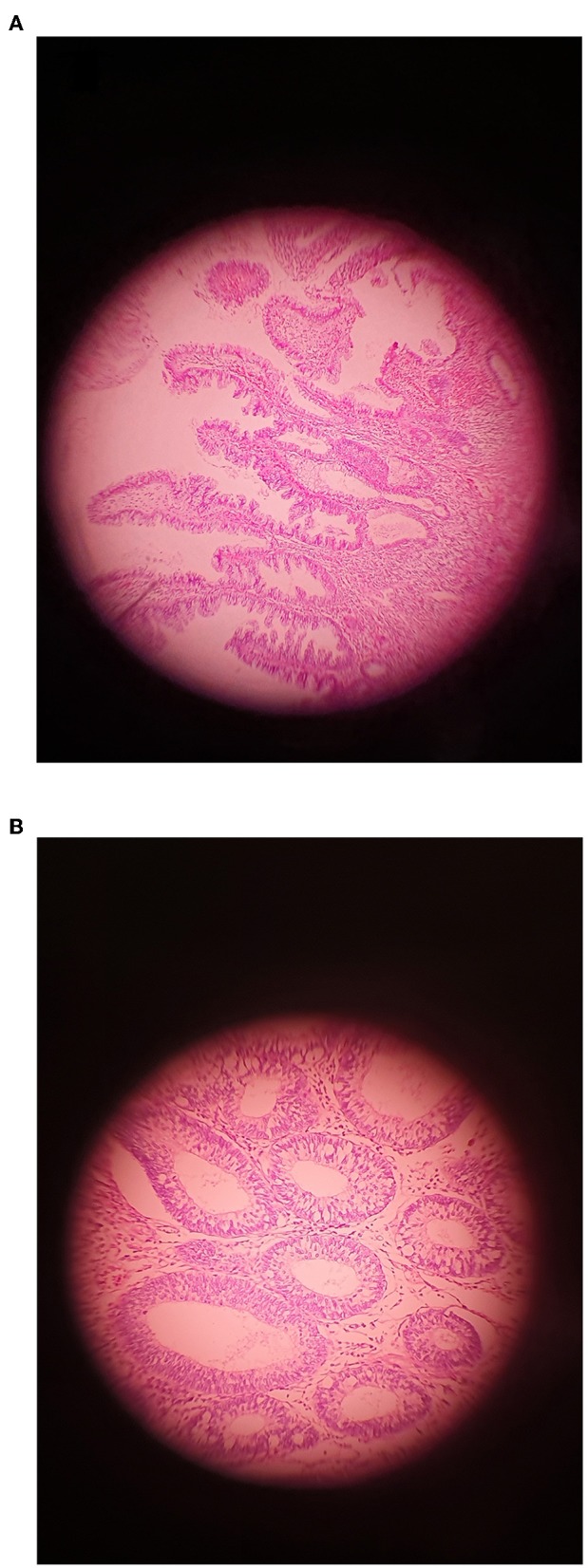
An example of Adenoma showing **(A)** LGD and **(B)** HGD (Original magnification ×400).

In this study, 68 samples were collected from CRC patients (36 males and 32 females; mean age: 55 yrs.; range: 35–78) and 52 samples were taken from healthy controls (HC) (30 males and 22 females; mean age: 56 yrs.; range: 42–78). All of the patients diagnosed based on colonoscopies procedures at the time of sampling. All of the patients diagnosed at the time of colonoscopy giving a total of 26 of patients with invasive CRC, 18 of patients with serrated lesions, 24 patients with adenoma include 14 patients with LGD and 10 of patients with HGD as per current classification (Figure 1). These biopsies were collected from right-side (ascending), left-side (descending), and both sides in 22, 32, and 14 patient, respectively (Table 1).

**Table 1:** Patients and HC characteristics.

		**Patients**	**Healthy controls**
	**Age range**	**35–78 years**	**42–78 years**
		**Number (%)**	**Number (%)**
Gender	Male	36 (53)	29 (56)
	Female	32 (47)	23 (44)
Colorectal lesion		68 (100)	52 (100)
	Serrated lesions	18 (26)	
	Adenoma with LGD^*^	14 (20)	
	Adenoma with HGD^**^	10 (14)	
	CRC^***^	26 (38)	
Sites of lesions			
	Ascending colon (Right side)	22	
	Descending colon (Left side)	32	
	Both	14	

* *LGD, Low-grade dysplasia*.

** *HGD, High-grade dysplasia*.

*** *CRC, Colorectal cancer*.

The results for *B. fragilis* culture was positive for 31(51%) and 25 (48%) samples of the patients and healthy controls, respectively (Table 2) (*P* = 0.7).

**Table 2:** Data on *Bacteroides fragilis* culture.

	**Culture**		**Total**
	**Negative** ***n* (%)**	**Positive** ***n* (%)**	
Healthy control	27 (52)	25 (48)	52 (100)
CRC	36 (53)	32 (47)	68 (100)
*P value*	0.7		

According to the standard curve, dilutions of ETBF (control positive) DNA at 10^1^, 10^2^, 10^3^, 10^4^, 10^5^, and 10^6^ provided Ct values of 15.08 ± 0.1, 18.04 ± 0.2, 21.42 ± 0.2, 25.02 ± 0.2, 28.02 ± 0.4, and 31.96 ± 0.4, respectively. The efficiency of the real-time PCR was between 98 and 100%.

Positive samples for 16S rRNA gene and *bft* gene were 63 and 47% in patients. However, positive results were shown in 81 and 3.8% of HC samples, respectively (Table 3). The difference between the positivity of *bft* gene in patients and HC was statistically significant (*P* = 0.00).

**Table 3:** The number of positive samples for 16S rRNA gene and *bft* Genes in clinicopathological groups and HC.

		***B. fragilis***	**ETBF**	
	***n***	***n* (%)**	***n* (%)**	**OR, 95% CI**
Healthy controls	52	42 (81)	2 (4)	
Serrated lesions	18	12 (67)	10 (55)	OR 31.25, 95% CI: (5.76, 169.65)
LGD	14	10 (71)	7 (50)	OR 25, 95% CI: (4.3, 145.21)
HGD	10	6 (60)	4 (40)	OR 16.67, 95% CI: (2.5, 111.08)
CRC	26	15 (58)	11 (42)	OR 18.33, 95% CI: (3.65, 92.03)
All patients	68	43 (63)	32 (47)	OR 22.22, 95% CI: (5, 98.74)

Also, the number of *bft* genes positive samples in CRC and HC within clinicopathological groups is shown in Table 3. The highest OR was found in serrated lesions group followed by adenomatous lesions with LGD group. Also the OR for all of the patients (OR 22.22, 95% CI: 5, 98.74) described the association of ETBF and the existence of lesions.

The results of the quantitative analysis of real-time PCR for 16S rRNA gene counted for *B. fragilis* and *bft* gene counted for ETBF per ng DNA were shown in Table 4. The difference between the copy numbers of 16S rRNA gene in patients and HC was not statistically significant (*P* ≥ 0.05). The copy numbers of *bft* gene were more in the samples of patients than in those of healthy controls (*P* = 0.00). Moreover, the copy numbers of *bft* gene were more in ETBF positive samples in the precursor lesions group compared to those with CRC; however, this difference was not statistically significant. The sequencing results confirmed the presence of *bft* and 16S rRNA genes (Data Sheet 1).

**Table 4:** Quantitative analysis of the 16S rRNA gene and *bft* genes in clinicopathological groups and HC.

		**Copy number of ETBF/ng DNA**		**Copy number of** ***B. fragilis*****/ng DNA**	
	***n***	**Mean**	SEM^*^	**Mean**	**SEM**
Healthy controls	52	0.03	0.03	4.1 × 10^2^	199.9
all patients	68	6.51 × 10	17.5	1.35 × 10^2^	40.8
Serrated lesions	18	1.2 × 10^2^	12.1	1.5 × 10^2^	32.1
LGD	14	1.3 × 10^2^	15.2	1.7 × 10^2^	42.6
HGD	10	1.2 × 10	14.3	8.2 × 10	54.1
CRC	26	1.8 × 10	18.8	1.9 × 10^2^	36.5

* *SEM, std. error of mean*.

Moreover, the results of these samples indicated that the most prevalent subtype of *bft* gene was *bft1* followed by *bft2*. From 32 *bft* gene harboring isolates, 18 (56.2%) isolates subtyped as *bft-1* and 14 (43.7%) isolates subtyped as *bft-2*. In addition, the *bft* gene was detected in 2 of HC samples, and all of them were subtyped as *bft-1*. No DNA sample harbored the subtype *bft-3*.

## Discussion

There is growing evidence for the effect of microbial dysbiosis in the gut, initiation, and development of colorectal cancer (Sears and Garrett, 2014; Gagniere et al., 2016). Although the incidence of CRC was reported lower in Iran compared to other countries, its rate was anticipated to increase in the future (Hosseini et al., 2004; Malekzadeh et al., 2009). In previous studies, it was also reported that ETBF may have a role in diarrhea and IBD (Myers et al., 1987; Prindiville et al., 2000; Basset et al., 2004; Merino et al., 2011; Purcell et al., 2017; Zamani et al., 2017).

Some investigations have suggested that certain bacterial species (e.g., ETBF) may operate as pathogenic bacteria that clarify the development of dysbiosis in microbial community of the gut and trigger CRC (Hajishengallis et al., 2012; Hajishengallis and Lamont, 2016). The colorectal tumorigenesis was induced by immune responses and activation of proinflammatory cytokines due to BFT (Wu et al., 2004, 2009).

The findings of this study indicated that ETBF was significantly associated with the serrated lesions followed by LGD. The higher odds ratio in these lesions also showed that ETBF exposure is a risk factor for the cancerous and especially precancerous states. This supports the hypothesis that BFT producing strains may have an important role in triggering the inflammation and immunological response in genetically susceptible persons and may lead to CRC. The first study demonstrating an increased prevalence of ETBF in the stool specimens of colorectal cancer patients (38%) compared with the control group (12%) conducted by Ulger Toprak et al. (2006). Moreover, some previous reports have shown an association between *bft* gene and CRC, particularly in the late stage of CRC (Dejea et al., 2014; Boleij et al., 2015; Viljoen et al., 2015). A study conducted by Purcell et al. demonstrated significant associations of ETBF with tubular adenomas, serrated lesions, and low-grade dysplasia, which was similar to the results of the present study (Purcell et al., 2017).

In this study, precursor lesions, including those with low-grade dysplasia, showed an increasing trend in *bft* gene amount compared to those with CRC; however, this difference was not statistically significant. Moreover, the authors have previously reported that ETBF markers were observed in the colon and terminal ileum of the ulcerative colitis patients who are predisposed to CRC (Zamani et al., 2017; Rashidan et al., 2018). Similarly, in a previous study, only *bft1* subtype of this gene was detected (Zamani et al., 2017). Although *bft1* gene was the most prevalent subtype, the *bft2* gene was also found in this study.

In this study, ETBF was detected in the lesions of the CRC patients, and similar results have also been previously reported by other investigators who studied ETBF in colonic mucosal samples (Boleij et al., 2015; Viljoen et al., 2015). Recent studies on the role of the gut microbiome have demonstrated that dysbiosis in the microbial community occurs in the non-tumor and tumor regions of the CRC patients (Dejea et al., 2014; Flemer et al., 2017; Purcell et al., 2017). In these studies showed that some of the bacteria including *Fusobacterium nucleatum*, ETBF, *Escherichia coli, Streptococcus gallolyticus*, and *Enterococcus faecalis* and butyrate-producing bacteria may play important roles in the development of CRC (Dejea et al., 2014; Flemer et al., 2017; Park et al., 2018).

The results of Real-time PCR for detection of *B. fragilis* showed that more percentage of the patients and HC contained *B. fragilis* compared to the culture method. The gold standard method for detection of bacteria is culture-based but it requires a high number of viable cells and specifically for anaerobic bacterial culture, some limitations could be occurs. May be these limitation affect the difference between the results.

The differences in *B. fragilis* cultures and Real-time PCR results between controls and CRC samples is not significant. These data suggest that probably the strains that harbor *bft* gene, not *B. fragilis* by itself, could contribute to CRC in this study. It has been shown that *B. fragilis* is a prominent human commensal, so this bacterium can be isolated from healthy controls like the patients too. But in the patients with cancerous and precancerous lesions some of the strains that contains *bft* genes increased and this dysbiosis likely can induce inflammation. Additionally some studies have shown that the human commensal *B. fragilis* prevented the development of colitis and may provide an effective therapeutic strategy for CRC while various studies suggest that enterotoxigenic strains of this bacterium is associated with intestinal tumors due to enterotoxin production (Sears et al., 1995, 2014; Mazmanian et al., 2008; Lee et al., 2018).

There were some limitations in this study. Different methods were used to diagnose the *bft* gene, which was extracted directly from tumor tissues in the colon of the patients. Thus, conducting a large population-based cohort study is highly recommended. Moreover, further investigations are needed to prove a possible correlation between the presence of *bft* gene and serrated lesions, LGD, and CRC.

## Conclusions

Results of this study suggest that ETBF could be present in the mucosal biopsies of the patients with precancerous conditions, such as serrated lesions and LGD, in addition to CRC patients. A significant association was found between the presence of ETBF in the affected tissues and the number of these bacteria in the samples of the patients, especially in the precancerous carcinogenic lesions: adenomas with low-grade dysplasia and serrated lesions. In fact ETBF is more often detected in early lesions but further research with higher number of specimens seemed to be helpful to determine precisely.

Also additional research is necessary to determine whether age, sex, diet, and other environmental factors affect ETBF diagnosis in humans over time.

Finally, ETBF could be a marker of CRC prognosis, particularly in the precancerous lesions, and could be used to screen these disorders.

## Data Availability Statement

The raw data supporting the conclusions of this manuscript will be made available by the authors, without undue reservation, to any qualified researcher.

## Ethics Statement

The studies involving human participants were reviewed and approved by Ethics Committee of Tehran University of Medical Sciences. The patients/participants provided their written informed consent to participate in this study.

## Author Contributions

SZ is the first author who performed all laboratory experiments, collected and analyzed data, and drafted the manuscript. RT is the gastroenterologist who did colonoscopy and provided the specimens from all cases. LS participated in the study design and coordination and advised in all parts of the study. AS and SJ participated in collecting the samples and performing the tests. MF has supervised all parts of the study. All authors read and approved the manuscript.

### Conflict of Interest

The authors declare that the research was conducted in the absence of any commercial or financial relationships that could be construed as a potential conflict of interest.

## Supplementary Material

Data Sheet 1 The sequencing results of *bft* and 16S rRNA genes in one isolate.
