# Supplementary material for: Enterotoxigenic Bacteroides fragilis: A Possible Etiological Candidate for Bacterially-Induced Colorectal Precancerous and Cancerous Lesions
Source: Front Cell Infect Microbiol. 2020 Jan 17;9:449. doi: 10.3389/fcimb.2019.00449 (PMC6978650; doi:10.3389/fcimb.2019.00449)
Supplement: Data Sheet 1 — The sequencing results of bft and 16S rRNA genes in one isolate. [file Data_Sheet_1.PDF]

Bft gene Seq Isolate 10
AAATGAAGTTAGTGCCCAGATGCAGGATGCCCTAAACTCGGTTTATGCAGTTCATGGACTGAAAAGATATGTC
AATTTCCACTTTGTACTGTATACTACTGAATACAGTTGTCCAAGTGGCGACGCCAAAGAGGGACTGGAAGGCT
TTACTG

16s rRNA gene Seq Isolate 10
CACTGGACTGCAACTGACACTGATGCTCGAAAGTGTGGGTATCAAACAGGATTAGATACCCTGGTAGTCCACA
CAGTAAACGATGAATACTCGCTGTTTGCGATATACAGTTAGCGG
